# The first report of porcine parvovirus 8 (PPV8) on the American continent is associated with pigs in Colombia with porcine respiratory disease

**DOI:** 10.1007/s00705-024-06099-z

**Published:** 2024-08-16

**Authors:** Diana S. Vargas-Bermudez, Jairo Jaime

**Affiliations:** Universidad Nacional de Colombia, Sede Bogotá, Facultad de Medicina Veterinaria y de Zootecnia, Centro de Investigación en Infectología e Inmunología Veterinaria (CI3V), Carrera 30 # 45-03, Bogotá, D.C Colombia

**Keywords:** Porcine parvovirus 8 (PPV8), Porcine parvoviruses (PPVs), Novel porcine parvovirus (nPPVs), Porcine respiratory disease (PRD), Coinfections

## Abstract

Seven novel porcine parvoviruses (PPV2 to PPV8) have been discovered in the last two decades. The last one reported was PPV8 in China in 2022, which was proposed to be a member of the genus *Protoparvovirus*. Here, we report the first detection of PPV8 outside China – in two provinces from Colombia. Six out of 146 (4.1%) pigs showing porcine respiratory disease (PRD) tested positive for PPV8. Sequencing and phylogenetic analysis of two Colombian PPV8 isolates (GenBank database accession numbers PP335559 and PP335560) showed them to be members of the genus *Protoparvovirus*. Furthermore, PPV8 was detected in coinfections with porcine circovirus type 2 (PCV2) and porcine reproductive and respiratory syndrome virus (PRRSV), which are associated with PRD.

**Supplementary Information:**

The online version contains supplementary material available at 10.1007/s00705-024-06099-z.

Porcine respiratory disease complex (PRDC) is a major cause of production losses in the global swine industry. This disease has a multifactorial origin and results from infection with different combinations of pathogens (including viruses, bacteria, and parasites), in addition to non-infectious factors such as environmental conditions, the age of the animal, population size, management strategies, and genetics [1]. The causative viral agents of PRDC include primary (putative) pathogens such as porcine reproductive and respiratory syndrome virus (PRRSV), porcine circovirus type 2 (PCV2), swine influenza A virus (IAV), and Aujeszky’s disease virus (ADV). In addition, the involvement of other viruses, such as porcine orthopneumovirus (SOV), porcine parainfluenza virus 1 (PPIV1), porcine circovirus 3 and 4 (PCV3, PCV4), porcine respiratory coronavirus (PRCV), porcine cytomegalovirus (PCMV) [2], and some novel parvoviruses (nPPV), has been suggested [3].

Parvoviruses (PVs) are non-enveloped viruses with a linear ssDNA genome. They belong to the family *Parvoviridae*, which includes viruses that infect invertebrates and vertebrates [4, 5]. In recent years, due to advances in high-throughput sequencing techniques and metagenomic analysis, novel parvoviruses have been discovered in various animal species [5]. Eight porcine parvoviruses (PPVs), designated as PPV1 through PPV8, have been discovered in the swine population [6–11]. PPV1 has been reported since the 1960s and is considered the oldest of the PPVs. It is associated with porcine reproductive failure (PRF) and causes different clinical manifestations that are collectively named “SMEDI” (stillbirth, mummification, embryonic death, and infertility) [12]. Seven nPPVs, from PPV2 to PPV8, were discovered in the last decades, with some proposed to be associated with clinical syndromes [3]. PPV2 was first detected in 2001 [13] and is currently the nPPV most frequently found in pigs with PRDC, with a prevalence reaching 75% [14]. In addition to the above and due to its detection by in situ hybridization (ISH) in the lung tissue of pigs suffering from respiratory failure (unpublished data), it has been suggested as a potential pathogen of PRDC [15, 16]. PPV3 was first reported in 2010 in Hong Kong in samples collected in slaughterhouses [6] and was later found distributed on various continents [17–20]. Regarding its association with PRDC, PPV3 has been detected at high viral loads in the lungs of pigs with respiratory disease [21]. PPV4 was first reported in 2010 in lung samples of pigs in the USA [22] and has also been detected in lungs with edema [14]. PPV5 was initially reported in the USA in 2013 in pig lungs [23], but its possible association with PRDC has not been established. PPV6 was first reported in China in 2014 in cases of abortions and stillbirths in herds experiencing PRF, as well as in healthy herds [8]. PPV6 is also one of the nPPVs that is detected most frequently in the lungs of pigs with PRDC [24]. PPV7 was discovered in 2016 in the USA through metagenomic analysis of stool samples [10], and its possible association in pigs with PRDC is yet to be established. Finally, PPV8, the most recent nPPV, was reported for the first time in China in September 2022 [11]. It was identified via high-throughput sequencing (HTS) in pig lung samples collected in 2021 in Guangdong province. In the same study, PPV8 was detected in samples of various tissues stored between 1990 and 2021 and found together with PRRSV in lung tissue. Those investigators determined the nearly complete genome (NCG) sequence of one PPV8 isolate, and that sequence is currently still the only one available. The PPV8 NCG is 4,380 nucleotides (nt) in length and has two overlapping open reading frames (ORFs), encoding the proteins non-structural 1 (NS1) and viral protein 1 (VP1), respectively. Phylogenetic analysis revealed that PPV8 was segregated in a different clade from other members of the genus *Protoparvovirus.*

In the present report, lung tissue samples that were collected during 2021–2022 from 76 swine herds located across Colombia´s five major swine-producing provinces – Antioquia, Atlántico, Valle de Cauca, Eje Cafetero, and Cundinamarca – were examined (Fig. 1). These samples were collected as part of a previous study aiming to determine the presence or absence of viruses implicated in PRDC, such as PCV2, PCV3, and PRRSV. Additionally, as a scientific interest, we routinely tested all samples received for some of the viruses that have been reported recently in the literature, including PPV8 and PCV4, which have never been reported in Colombia. As the original objective was the detection of viruses involved in PRD, samples were collected only from cases of pigs with respiratory clinical signs (dyspnea, tachypnea, and cyanosis). A total of 146 lung tissues were collected from nursery pigs (*n* = 122; aged 3 to 8 weeks) and grow-finisher pigs (*n* = 24; aged 8 to 23 weeks). Lung tissues were minced and diluted at 1:10 (weight/volume) in PBS (pH 7.4), homogenized, and centrifuged at 1,500 × *g* for 10 min. Total viral nucleic acids were extracted from 200 μL of the supernatant using a High Pure Viral Nucleic Acid Kit (Roche, Mannheim, Germany), following the manufacturer’s instructions. All extracts were subsequently stored at -80°C until used for molecular analysis. The initial testing for PCV2, PCV3, and PRRSV was carried out via real-time PCR using TaqMan probes, employing specific primers and protocols that were reported previously [25]. Among these viruses, PRRSV was the most prevalent, detected in 65% (96/146) [95% CI; 57.2 − 72.7%] of the samples, followed by PCV2 in 63% (93/146) [95% CI; 53.0–70.8%] and PCV3 in 24% (36/146) [95% CI; 17–30.9%]. PPV8 was detected using conventional nested PCR (nPCR), employing specific primers as reported previously by Guo et al. [11]. The first amplification was performed using the primers PPV8-outF (5′-TGTTGGTTTGCACCTAGCG-3′) and PPV8-outR (5′-TGATGAGATGGTGGAACGC-3'). The second amplification was performed using the primers PPV8-inF (5′-TCCAAGTTGCCCTAGACAGC-3′) and PPV8-inR (5′- GCCTCGTACATGTGGACCTC-3′), producing a final product of 554 bp. Reactions were carried out in a total volume of 25 μL comprising 0.25 μL of Taq polymerase (5 U/μL) (Go Taq Flexi-Promega), 2.5 μL of 5x Taq buffer, 1 μL of each primer (20 μmol/μL), and 2 μL of extracted DNA. The thermal cycling conditions for each PCR included an initial denaturation step at 94°C for 5 min, followed by 35 cycles of denaturation at 94°C for 30 s, annealing at 58°C for 30 s, and extension at 72°C for 30 s, and a final elongation step at 72°C for 10 min. The specificity of the assay was confirmed by testing DNA extracted from previous samples that were positive for other DNA viruses, including PCV2, PCV3, and PPV1 to PPV7. In this study, PPV8 was detected in 4.1% (6/146) [95% CI; 0.8– 7.3%] of the total lung samples tested (*n* = 146). At the herd level, the virus was detected in 3.1% (3/76) [95% CI; 0.4–8.2%] of the herds tested, and by age groups, it was detected in 3.3% (4/122) of nursery pigs and 8.3% (2/24) of grow-finisher pigs. By geographical regions, PPV8 was detected in two provinces (Cundinamarca and Eje Cafetero) out of the five surveyed. Comparing our study with the only other study in which PPV8 has been detected [11], we found a coincidence in the sample selection criteria since the pigs in both studies were sick with respiratory symptoms. Our sampling was larger (*n* = 146) versus (*n* = 55), and we covered the five provinces with the highest pork production in Colombia. In contrast, in the study from China, only one province (Guandong) was evaluated. Our study is the first to provide an approximation of the prevalence of PPV8 by tissue (lung) and groups evaluated (nursery and grow-finisher pigs) as well as by geographic distribution. In the study from China, PPV8 was detected in seven out of in 7/55 PRRSV-positive lungs, corresponding to a prevalence of 12.7%, which is higher than that found in Colombia (4.1%). An important aspect to discuss is the tropism of PPV8. Although these two studies reflect tropism for lung tissue, other studies [3, 16, 26] have shown that other nPPVs (PPV2 to PPV7) have been detected in different samples and tissues In the Chinese study [11], PPV8 was detected in 37 stored tissues collected between 1998 and 2021, with lung, kidney, spleen, liver, and lymph nodes testing positive. A major limitation of our study is that only lung tissues were tested. Therefore, it is still necessary to test for PPV8 in other organs and to determine whether it is associated with clinical manifestations. It is notable that all of the lung samples from the present study were negative for PCV4.

**Fig. 1:**
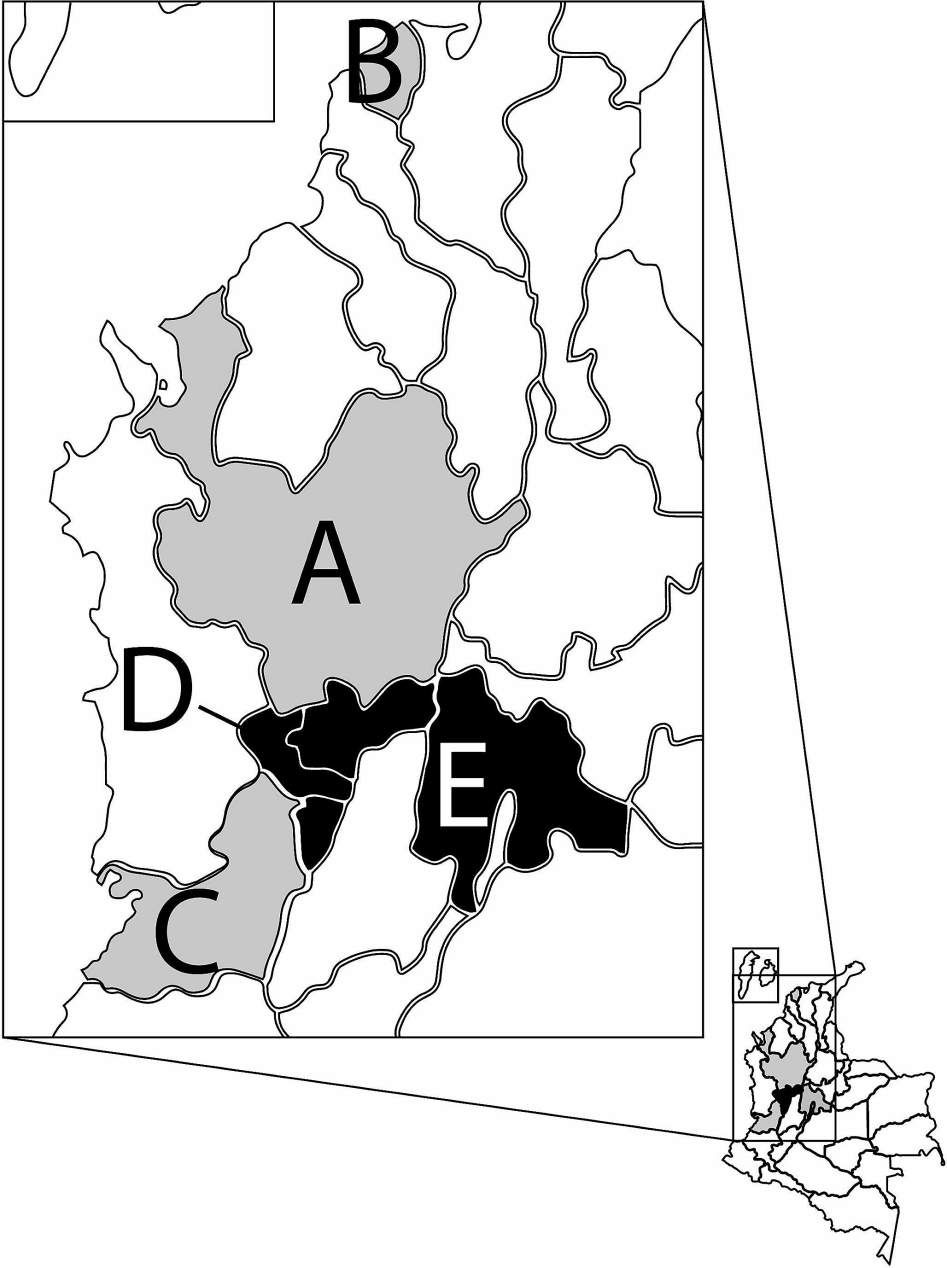
Map of Colombia highlighting the five provinces selected for this study, which have the highest level of pig production in the country. (**A**) Antioquia. (**B**) Atlántico. (**C**) Valle del Cauca. (**D**) Eje Cafetero. (**E**) Cundinamarca. The provinces (**D** and **E**) where PPV8 was detected are highlighted in black. The map was created using the National Department of Statistics of Colombia (DANE) database (https://geoportal.dane.gov.co/acerca-del-geoportal/acerca/#gsc.tab=0) (accessed on 02 May 2024) and adapted using QGIS software version 3.34.5, available online: https://qgis.org/es/site/ (accessed on 02 May 2024).

A sequencing strategy using partially overlapping primer pairs to determine the sequence of the coding region of the PPV8 genome. Primers were designed based on the PPV8 sequence from China (GenBank database accession number GDJM2021) using Primer 3 Input (v3.0.0; Institute for Biomedical Research, Boston, MA) [11]. A list of the primers used, some of which were described previously, and their target regions on the PPV8 genome is shown in Table 1. This resulted in a nearly complete genome sequence lacking the two inverted terminal repeats (ITRs). PCR reactions for sequencing PPV8 were carried out in a total volume of 25 μL containing 0.25 μL of AccuPrime Taq polymerase (5 U/μL) (Thermo Fisher), 2.5 μL of 1x AccuPrime PCR buffer, 1 μL of each primer (20 μmol/μL), and 2 μL of extracted DNA. PCR conditions comprised initial denaturation at 94°C for 2 min, followed by 35 cycles of denaturation at 94°C for 30 s, annealing at 57°C for 30 s, and extension at 68°C for 1 min. All reactions were performed in a C1000 Touch thermocycler (Bio-Rad, Foster City, California, USA). The PCR products were subsequently purified using a QIAquick PCR Purification Kit (QIAGEN) following the manufacturer’s instructions.

**Table 1:** Primers used in the present study for sequencing the PPV8 genome

Primer	Sequence (5´- 3´)
PPV8-225F	GGATGTCTTGACTTACAGATGGC
PPV8-1215R	TGAAATGGATGAAGGAGTCTGG
PPV8-1190F	AGATCCAGACTCCTTCATCCA
PPV8-2156R	TGTTTTCTTGCTGCAGCGTC
PPV8-1944F	GGAGAGCCATGGACAACCAA
PPV8-2952R	CATGCTGTCGCTGTCTAGGG
PPV8-2924F*	TCCAAGTTGCCCTAGACAGC
PPV8-3477R*	GCCTCGTACATGTGGACCTC
PPV8-3475F	GGCGTTCCACCATCTCATCA
PPV8-4338R	CCAAGACACCCAAGAGCGTT

*Primers PPV8 2924F and PPV8 3477R were reported previously [11].

Bidirectional Sanger sequencing was performed by a professional service (SSiGMol - Sequencing and Molecular Analysis Service, Institute of Genetics, National University of Colombia, Bogotá Campus). From the six PPV8-positive lung samples, sequences of two NCGs (4095 nt) were obtained and compared with those of PPV reference strains. Phylogenetic analysis was performed using our two PPV8 NCG sequences, the PPV8 sequence from China, and 39 PPV (PPV1 through PPV7) reference sequences obtained from the NCBI GenBank public database. These sequences were selected using nucleotide BLAST (Basic Local Alignment Search Tool), to include isolates from different continents. The sequences were aligned using MAFFT with default settings [27]. The two Colombian PPV8 NCG sequences (nt 243 to 4333), which included the VP and NS genes, have been deposited in the GenBank database under the accession numbers PP335559 and PP335560. The nt and aa sequence identity among two Colombian PPV8 sequences and the Chinese sequence (GenBank database accession number GDJM2021) ranged from 99.6–99.8%. The ORF1 region of the two Colombian sequences is 1,806 nt long and encodes an NS protein of 601 aa. The nt sequence identity of ORF1 ranged from 99.5–99.8%, and the aa sequence identity was 99.8%. The ORF2 of both Colombian sequences was 2,106 nt in length, encoding a 701-aa VP protein. The nt sequence identity among the ORF2 sequences ranged from 99.6–99.8%, and the aa sequence identity was 99.8%. Phylogenetic analysis was performed using MEGA 7.0 for Windows [12] by the maximum-likelihood (ML) method, with 1,000 ultrafast bootstrap replicates, and the Tamura 3 parameter model with gamma-distributed heterogeneity (T92 + G) nt substitution model was chosen as the best fit. The resulting tree (Fig. 2) revealed that the Colombian isolates (PPV8/Col/Cundinamarca3.19/2021 and PPV8/Col/E.cafetero4.1/2021) clustered together with the PPV8 isolate from China in an independent branch and were included in a clade with other members of the genus *Protoparvovirus.* The three PPV8 sequences shared the most sequence similarity with porcine bufavirus and were more distantly related to reference strains of PPV1. Based on its phylogenetic relationships, PPV8 is proposed to be a member of the genus *Protoparvovirus*, but its taxonomic position within the genus has yet to be defined.

**Fig. 2:**
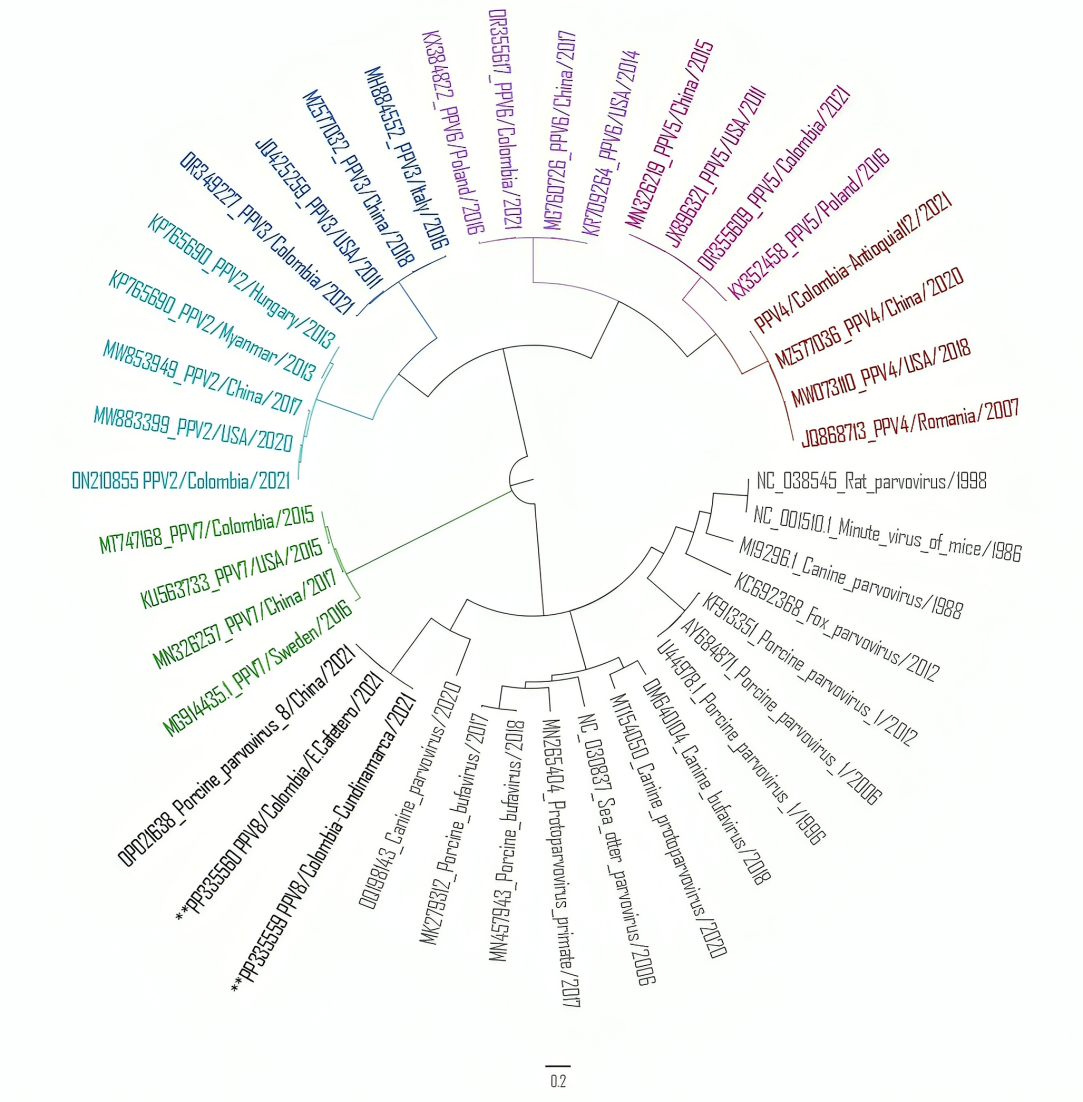
Maximum-likelihood phylogenetic tree of PPVs (PPV1 through PPV8) based on an alignment of complete genome nucleotide sequences. Sequences in black are from members of the genus *Protoparvovirus*, including three PPV8 sequences (highlighted in black), and ** indicates the positions of the two Colombian sequences from the present study

PPV8 was found to be present in both single infections and coinfections with other viruses. PPV8 mono-infection accounted for 16% (1/6) of the PPV8-positive lung samples. A dual infection with PRRSV and PPV8 was found in two of the six positive samples (33.3%), and a dual infection with PCV2 and PPV8 (16%) was found in one. A triple infection with PCV2, PRRSV, and PPV8 was detected in two samples (33%). In the study from China [11], PRRSV/PPV8 coinfections were also detected in lung samples, and classical swine fever virus (CSFV)/PPV8 coinfections were detected in tissues stored between 1998 and 2021. Our study is the first to identity coinfections between primary PRDC viruses (PRRSV and PCV2) and PPV8 in lung tissue, confirming earlier suggestions that nPPV coinfections occur frequently in different tissues and organs [3, 25].

The results of this study reveal that the two Colombian PPV8 isolates and the Chinese isolate belong to the same genus as PPV1 and form a separate subclade from other protoparvoviruses. The presence of PPV8 in pigs with respiratory signs, particularly in coinfection with other viruses (PRRSV and PCV2), suggests the possible participation of PPV8 in PRDC, but this still needs to be investigated experimentally. As with the other nPPVs, it needs to be clarified whether PPV8 is part of the normal porcine virome and whether it plays a role in disease when present in coinfection with other pathogens.

## Electronic supplementary material

Below is the link to the electronic supplementary material

Supplementary Material 1
